# Scaling distributed training in rural primary care: a Philippine model for LMIC health systems

**DOI:** 10.3389/fmed.2025.1649367

**Published:** 2025-12-03

**Authors:** Aileen T. Riel-Espina, Anna Guia Orejola Limpoco, Reggiena Las Piñas Lachica, Renato Banez Bolo Jr., Daniel John Dineros Lachica

**Affiliations:** 1 Sorsogon Province-Wide Family and Community Medicine Practice-Based Residency Training Program, Department of Family and Community Medicine, Sorsogon Provincial Hospital, Sorsogon, Philippines; 2 Department of Family and Community Medicine, University of the Philippines - Philippine General Hospital, Manila, Philippines; 3 Department of Family and Community Medicine, College of Medicine, University of the Philippines Manila, Manila, Philippines; 4 Department of Family and Community Medicine, College of Medicine, Pamantasan ng Lungsod ng Maynila, Manila, Philippines; 5 Sorsogon Provincial Health Office, Sorsogon City, Province of Sorsogon, Philippines; 6 Sorsogon Provincial Hospital, Sorsogon City, Sorsogon, Philippines

**Keywords:** family community medicine, distributed training, health systems strengthening, rural health, remote supervision

## Abstract

Low- and middle-income countries (LMICs) continue to face persistent shortages of trained primary care providers in rural areas. This challenge is compounded by the lack of locally accessible residency training programs, which limits opportunities for physicians to develop the skills needed to serve these underserved communities. Consequently, physicians working in underserved municipalities leave their posts to pursue specialization, exacerbating the workforce shortage. In Sorsogon, a province in the Philippines, this gap is further compounded by the mismatch between existing training models being offered in urban hospitals with a curative, episodic-care orientation and the province’s need for a community-embedded program anchored on health promotion, disease prevention, and continuity of care. This community case study describes the design and implementation of the Sorsogon Province-wide Practice-Based Family and Community Residency Training Program (PBFCMRTP), a distributed, in-situ model co-developed by the Provincial Government of Sorsogon and the Philippine Academy of Family Physicians (PAFP). Grounded in the principles of the Universal Health Care (UHC) Law of the Philippines, the program enables rural physicians to undergo residency training while remaining in their practice sites—provincial and district hospitals, as well as rural health units—ensuring uninterrupted service delivery during training. Using a hybrid, spiral curriculum that combines digital learning classrooms, peer learning, integrated case discussions, periodic practice site visits, and workplace-based assessments, remote supervision with mentoring sessions by accredited family medicine trainers, the program emphasizes health systems integration, primary care leadership, and community-responsive care grounded on the Patient-Centered, Family-Focused, and Community-Oriented (PFC) approach. Over 4 years, the program has matured from a new program granted provisional accreditation status by the PAFP Residency Accreditation Board to full (Level 3) accreditation status. It has successfully prepared its trainees to lead primary care delivery in resource-constrained, community-based settings. This case highlights the feasibility of scaling practice-based residency training models in LMICs through strong local governance, policy support, and community-responsive curriculum design. Key enablers include the strategic use of digital platforms, trainees’ commitment, dedicated trainers, and intersectoral collaboration. Lessons from the Sorsogon experience may inform efforts to decentralize medical education and strengthen rural health systems in similar contexts.

## Introduction

Health workforce shortages in rural and remote areas persist as a significant barrier to achieving equitable and cost-effective healthcare, particularly in low-and middle-income countries (LMICs). Globally, the mismatch between where physicians are trained and where they are most needed continues to challenge the achievement of Universal Health Care (UHC). The World Health Organization (WHO) projects a shortfall of 18 million health workers by 2030, with rural populations in LMICs most severely affected. Traditional residency training models are typically hospital-based, curative in focus, and located in urban centers. They require physicians to relocate for training, contributing to workforce migration and further weakening of rural health systems. Furthermore, Immersive exposure to specialized, hospital-centric environments also shapes trainees’ long-term career preferences, making them more likely to remain in cities after graduation (1). In contrast, distributed training programs have shown promise; evidence from Canada suggests that residents trained in community-based sites are 15 to 36 times more likely to practice in rural areas than their urban-trained counterparts (2, 21).

The Philippines reflects many of these global trends. Despite national reforms under the UHC Law that mandate the assignment of a primary care provider for every Filipino, rural areas continue to face a chronic undersupply of trained and certified primary care physicians (20). Over 60% of Filipinos live in rural areas, yet the majority of physicians remain concentrated in urban centers due to a combination of professional, educational, lifestyle, and systemic factors. In these underserved areas, it is not uncommon for a single physician to be the sole healthcare provider in the community. Feelings of professional isolation, heavier workloads, and limited opportunities for career growth and professional development deter most physicians from pursuing long-term rural practice (19).

Sorsogon, a predominantly rural province in the southernmost part of Luzon, exemplifies this challenge. Despite aggressive local efforts—including active recruitment and expanding benefit packages—many young doctors leave after only 1 or 2 years of service to pursue residency training in urban centers due to the absence of accredited residency programs in the province. Those who remain often feel professionally stagnant, as the local health system cannot afford to release them for full-time training without compromising service delivery.

Compounding the geographic inaccessibility of residency training programs is the pedagogic misalignment of the traditional urban-based, hospital-centric model with the goals of UHC, which emphasize primary care, health promotion, and disease prevention. What Sorsogon needed was a training model that could capacitate rural physicians without requiring them to leave their posts, which was also aligned with the national goals for primary care oriented health systems.

This community case study documents a real-world, practice-driven innovation that emerged in response to the priorities of a local government unit. It examines the design, implementation, and early outcomes of the Sorsogon Province-wide Practice Based Family and Community Medicine Residency Training Program (PBFCMRTP)—a distributed, in-situ training model co-developed by the Provincial Government of Sorsogon and the Philippine Academy of Family Physicians. Anchored in principles of health systems integration and community-responsive care, the program aims to answer this question: *Can a locally designed and led, community-embedded residency program capacitate rural physicians to contribute meaningfully to health system strengthening, during and after training?*

## Context

**Figure 1:**
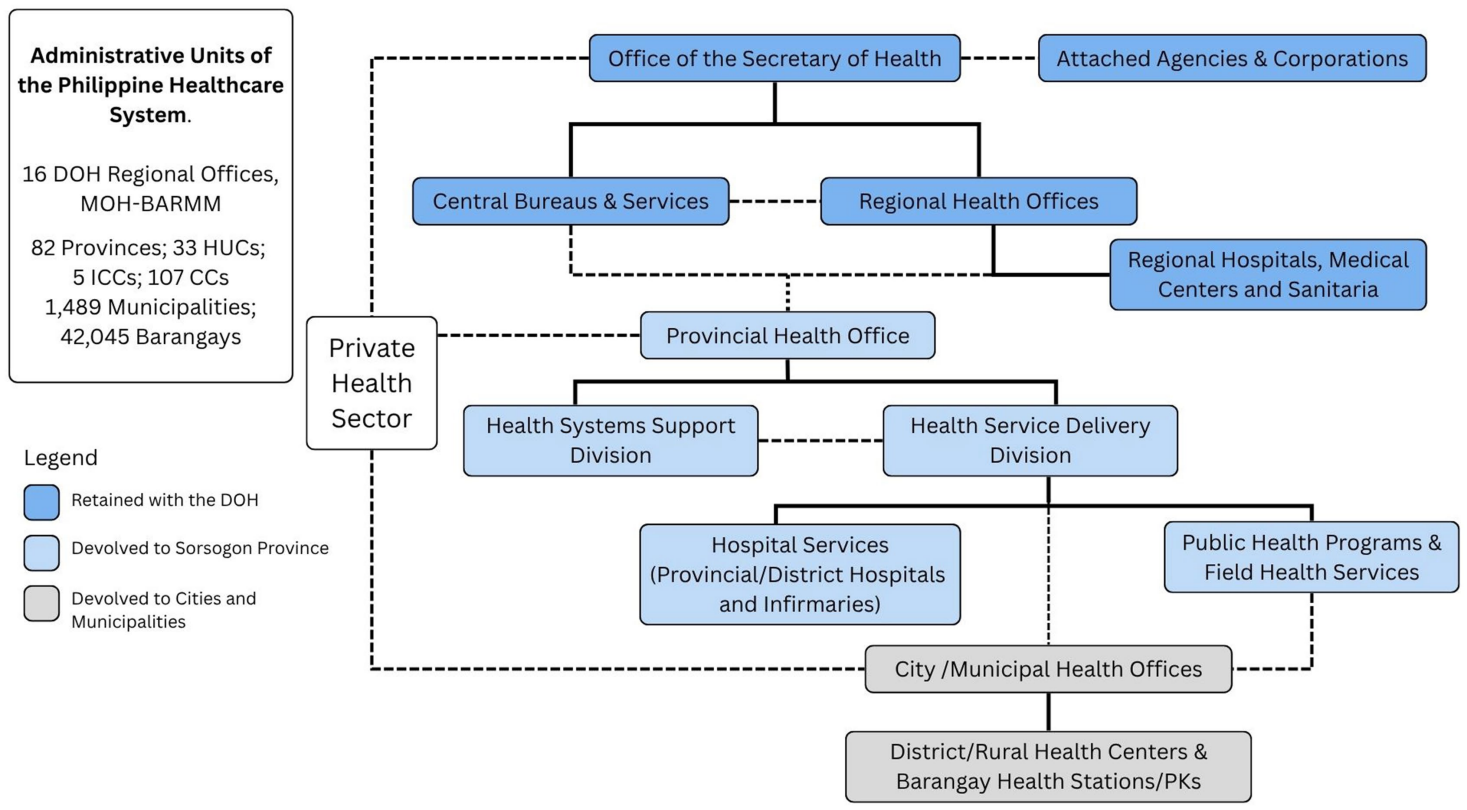
Organizational structure of the Sorsogon Provincial Health care System.

The Philippine health care system is a decentralized structure composed of both public and private sectors. Public health services are delivered through various levels of government: national (3–5), provincial, city, and municipal units, each managing corresponding hospitals and health offices. While the Department of Health retains oversight of regional hospitals and central services, most frontline services have been devolved to local government units (LGUs), creating a complex network of shared responsibilities across regions, provinces, cities, and municipalities (Figure 1).

Sorsogon is a predominantly rural province located at the southeastern tip of the Luzon island mass in the Philippines. It has a population of approximately 828,000 people distributed across one city and 14 municipalities, including geographically isolated and disadvantaged areas such as islands and coastal barangays. The provincial health system is organized into four inter-local health zones and comprises one provincial hospital, four district hospitals, four infirmaries, 17 municipal health offices or rural health units (RHUs), and over 200 barangay health stations (BHSs). Despite the presence of these facilities, the province continues to face significant challenges in delivering accessible, high-quality healthcare services, particularly in remote and underserved communities.

**Figure 2:**
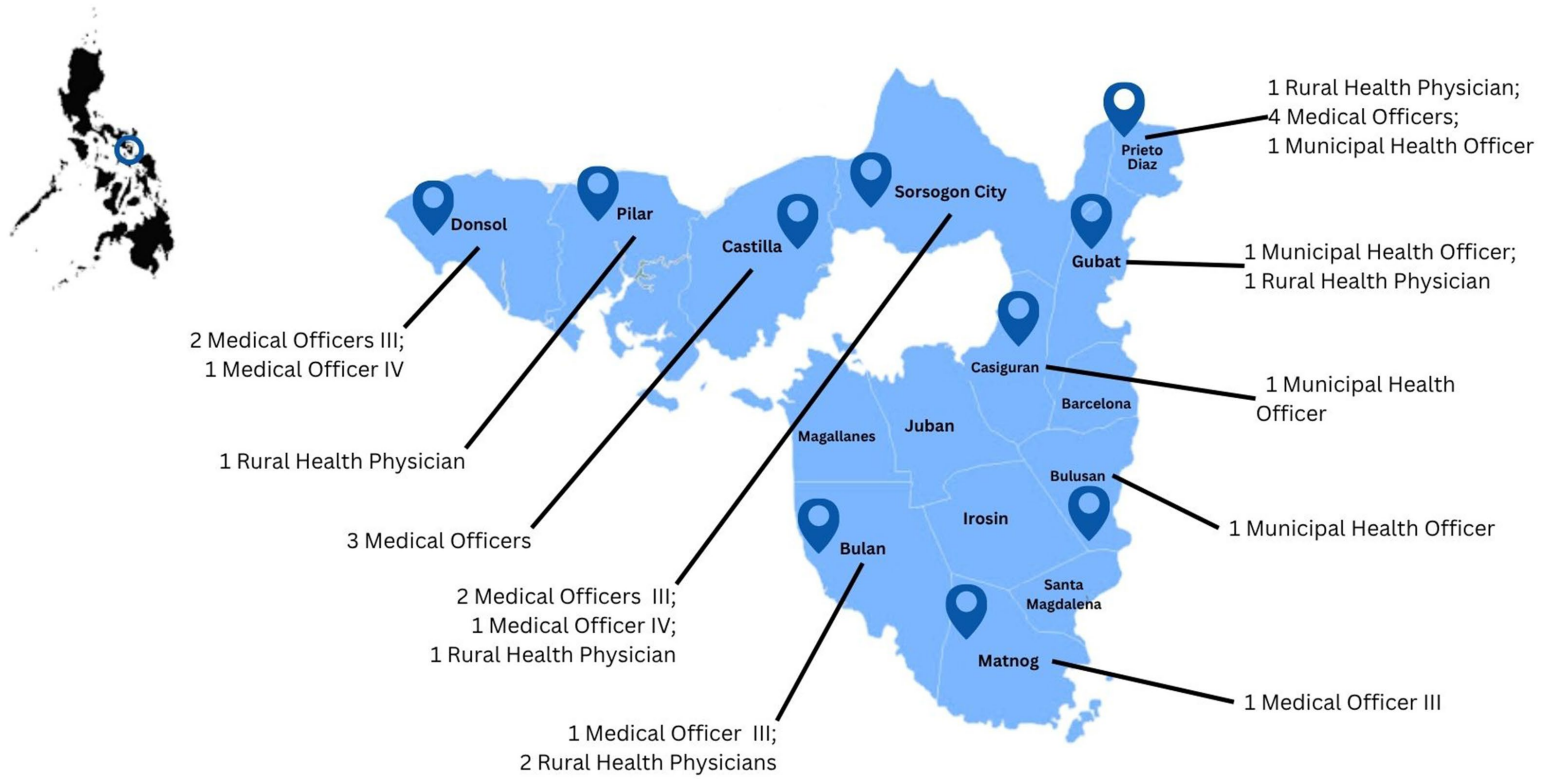
Geographic distribution of trainees throughout the province.

The shortage of qualified physicians, particularly family and community medicine specialists, has long hindered the province’s ability to fulfill its health service mandates and achieve its health objectives. Many rural health facilities operate with only one physician, often untrained or undertrained, who frequently serves as the sole provider for a wide catchment area, exceeding the ideal 1:10,000 doctor-to-population ratio. Turnover is high, with newly recruited doctors often leaving after a short period to pursue residency training in urban centers. Traditional hospital-based residency programs, located far from the province and structured around curative, specialist care, are misaligned with Sorsogon’s needs and the competencies required for rural primary care delivery (Figure 2).

Recognizing these systemic constraints, the Provincial Government of Sorsogon, in partnership with the Philippine Academy of Family Physicians (PAFP), launched the Province-wide PBFCMRTP in 2021. Practice-Based Family and Community Medicine Residency Training Program is a training program track that occurs while trainees are in their communities/areas of practice. This innovation was first introduced in 2000 by the PAFP to enable physicians desirous to specialize in family medicine but cannot leave their posts/practice to pursue traditional hospital-based training. It is now being implemented all over the country in various iterations with enrollees coming from different types of medical practice settings.

The PAFP’s Practice Based Program was specifically designed to allow the trainees’ practice site to be their learning site as well. It was anchored in Kolb’s Experiential Learning Theory, which postulates that knowledge is created through the processing and transformation of learners’ actual experiences (6). The PAFP ensures that programs are evaluated for quality improvement hence the CIPP model is used as the basis of evaluation for accreditation. The CIPP model takes into account context and process and is not only focused on outcomes like the traditional Kirkpatrick model (7).

For Sorsogon province, the initiative will provide a venue for rural physicians employed in their health facilities to enhance their competencies through processing of their experiences and anchoring their actual practice in theoretical frameworks supplanted by the latest clinical evidence from research, resulting in a more nuanced and contextualized patient care delivery. The training curriculum is grounded in the Patient-Centered, Family-Focused, and Community-Oriented (PFC) service delivery framework (8) and is delivered through a combination of blended learning strategies, remote supervision, and mentorship. The training is contextualized within the provincial health system but compliant with national specialty residency training program accreditation standards.

Sorsogon’s designation as a Universal Health Care Advanced Implementation Site, along with its high number of primary care facilities accredited by Philippine Health Insurance Corporation (PhilHealth) under the KONSULTA (*Konsultasyong Sulit at Tama*) program, made it an ideal setting for piloting a distributed training model. The program aims to address the dual challenge of rural health workforce shortages and misaligned training models, by offering a sustainable and scalable approach to capacitating primary care providers within their communities. It also aims to demonstrate how primary care strengthening through training and capacity building of frontline primary care physicians can improve gatekeeping within the provincial Health Care Provider Network (HCPN) and subsequently improve health outcomes and reduce the cost of healthcare services.

## Key programmatic elements

**Figure 3:**
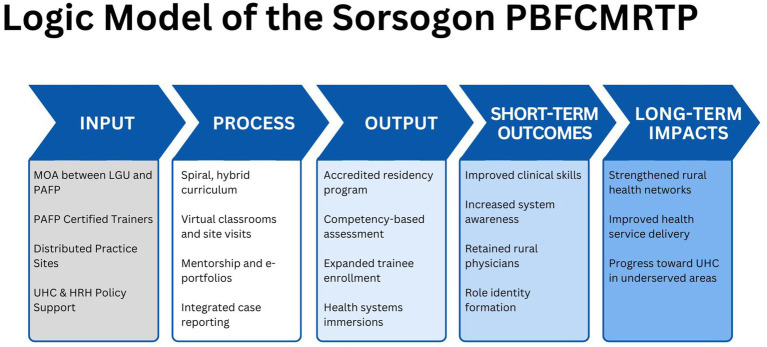
Theory of change for the Sorsogon Province-wide PBFCMRTP.

The Sorsogon PBFCMRTP is a province-wide distributed training model designed to strengthen the rural primary care workforce and improve service delivery. Its core objectives were to: (1) upskill rural physicians without disrupting service delivery, (2) align training with local health system needs, and (3) comply with national accreditation standards. The program followed the logic model shown in Figure 3 which illustrates how structured inputs and targeted activities in a distributed residency training program can generate educational outputs that will lead to system-wide outcomes and long-term health impacts. The approach aligns medical education with local service needs and national health reforms.

This design minimized service disruption and improved continuity of care, addressing both health workforce gaps and quality of care in underserved areas. These features align with global evidence on distributed medical education (9–11) which underscores the importance of training in the context of service.

### Fit-for-purpose curriculum design

The program employs a context-specific, ladderized curriculum aligned with the health needs of Sorsogon’s population and the service delivery goals of the provincial health system. It integrates national standards set by the PAFP, ensuring that trainees are eligible for specialty certification upon program completion. The curriculum is structured around the six core physician roles—clinician, educator, leader, researcher, navigator, and coordinator—applied progressively across four training levels. Table 1 below summarizes the expected competencies at each training level reflecting the program’s spiral, competency based curriculum.

**Table 1:** Competency progression across the training levels of the Sorsogon Province-wide PBFCMRTP as adapted from the PAFP toolkit for trainers (18).

Roles/Levels	Level 1	Level 2	Level 3	Level 4
Healthcare provider	Manage patients in ambulatory and urgent care settings independently	Discuss comprehensive health plans, recognize needs of special populations, refer patients appropriately, utilize clinical pathways, perform lifesaving procedures and manage patient documentation	Manage health concerns of special populations, perform limited advanced surgical skills, deliver population-based services, implement and monitor community-oriented care, and analyze disease patterns	Serve as a generalist and family medicine expert, uphold ethical standards, and engage in research (clinical and public health) and quality assurance activities
Researcher	Integrate case reports with evidence-based medicine appraisals	Apply evidence for clinical decision-making in acute, out and in-patient settings	Develop research protocols on health needs	Generate research to support quality care and inform policy
Educator/Counselor	Develop instructional designs for learning activities	Apply principles of team approach and demonstrate interpersonal skills	Create instructional designs for capacity building, use technology in education and train other members of the healthcare team	Contribute to shared knowledge and practice improving health outcomes
Care Coordinator	Conduct multidisciplinary meetingsfor shared decision making	Communicate effectively with families and health care teams and formulate proactive care plans	Efficiently utilize service delivery networks	Ensure continuity and safety of care across levels and networks
Patient Navigator	Facilitate referral of cases to relevant health and social programs	Manage patient transitions and identify health system partners for resource access	Strategize navigation processes and engage in shared decision making with other stakeholders	Facilitate access to health-related programs and resources using a whole of society approach
Leader/Manager	Participate in quality assurance activities and community-oriented primary care initiatives.	Design and implement communityoriented primary care action plans and activities	Evaluate and recommend improvement of community interventions	Demonstrate leadership and engage with community and healthcare networks

### Patient-centered, family-focused, community-oriented (PFC) framework

All learning and service delivery activities are anchored in the PAFP’s PFC service delivery framework. This model emphasizes holistic care that considers not only individual biomedical conditions but also family dynamics and community health determinants. The framework equips residents to deliver integrated care that is culturally responsive and aligned with public health goals.

### Distributed training sites

Training takes place across a network of district hospitals, rural health units, and barangay health stations throughout the province (Figure 2). This decentralized approach allows residents to remain embedded in their communities while developing competencies in real-world, resource-constrained settings. The distribution of training sites also facilitates integration into the province’s HCPN and service delivery network SDN.

### Blended learning and mentorship

The program uses hybrid instructional methods, combining face-to-face learning, virtual lectures, and self-directed modules delivered through digital platforms. Remote supervision and regular mentoring sessions by PAFP-accredited trainers support each trainee’s progression. The Integrated Case Management (ICR) method (12) is employed to develop competencies through real patient encounters, using the PFC framework, the six roles of Family Physicians and the use of evidence-based medicine.

### Health system integration

The Sorsogon Province-wide PBFCMRTP is intentionally designed to align with the goals of the UHC Law and the province’s designation as a UHC Advanced Implementation Site. It builds the capacity of primary care providers to function effectively within integrated health systems, improving gatekeeping, care coordination, and referral efficiency across levels of care.

The short term outcomes of the Sorsogon province wide PBFCMRTP demonstrated that high-quality postgraduate medical education can be delivered effectively through a distributed, practice-based model embedded within rural health systems. The program allowed rural physicians to continue serving in their localities while undergoing training contributing to health systems strengthening during training.

## Discussion

Community-embedded training initiatives, such as Australia’s Remote Vocational Training Scheme (RVTS) and the Targeted Medical Education Program (TMEP) in rural China, demonstrate the effectiveness of localized medical education in addressing health challenges in underserved regions. These programs emphasize practice-based training, remote supervision, and flexible learning tailored to the specific needs of the community. By fostering a connection to local health systems, these training models not only enhance the clinical competencies of healthcare providers but also contribute to the retention of physicians in rural areas, ultimately strengthening the overall health infrastructure (13–15).

The parallels between international models highlight the necessity for medical education that aligns with the realities of rural practice. Both the RVTS and the Sorsogon PBFCMRTP in the Philippines advocate for a curriculum that is responsive to local health needs, emphasizing continuity of care and public health challenges. A recent study in China underscores the gap between urban training and rural practice, reinforcing the demand for community-embedded, flexible education. These findings suggest that globally relevant residency programs, which integrate local health systems into their training frameworks, are essential for improving healthcare delivery in rural settings. The Sorsogon PBFCMRTP directly responds to these needs through a distributed, blended learning model with hybrid mentorship and a fit-for-purpose curriculum anchored in the PFC framework. These parallels affirm the global relevance and applicability of in-place rural residency programs tailored to local health systems.

The Sorsogon Province-wide PBFCMRTP has undergone a structured and progressive development since its inception. The journey began in March 2020 with the signing of a Memorandum of Agreement (MOA) between the PAFP and the Sorsogon Provincial Local Government Unit (PLGU), formalizing their partnership to co-develop a distributed residency training model anchored in primary care.

Following a year of preparatory work in the midst of the COVID-19 pandemic, the program officially launched in July 2021 with the enrollment of its first cohort of trainees. This marked the operationalization of the program’s innovative, in-situ training model, which utilized existing local government health facilities as the primary learning sites and available educational resources in the local community.

In March 2022, the program gained formal recognition from the PAFP as a new residency training program. A year later, in March 2023, it achieved Level 2 Program Accreditation, a milestone that affirmed the program’s adherence to core training standards and quality assurance mechanisms.

**Figure 4:**
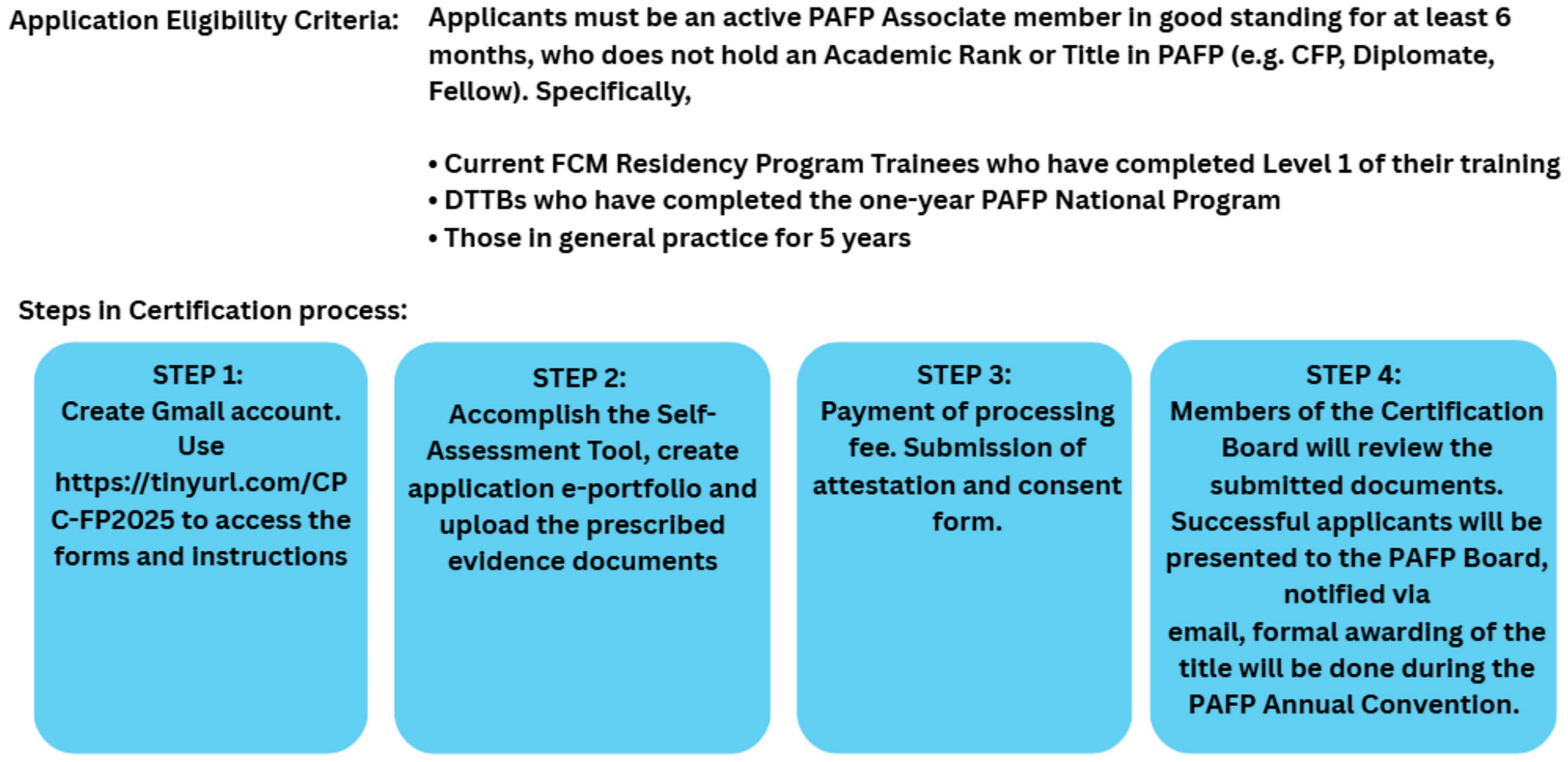
Certification process for the certified primary care family physician academic rank.

By March 2024, the program celebrated the certification of 14 trainees as Certified Primary Care Family Physicians (CPCFP), reflecting the effectiveness of its training approach and the competency of its product. This is the Academic Rank awarded to PAFP Members after successfully complying with the Certification Process (Figure 4) set by PAFP Primary Care Certification Board. Applicants were evaluated based on the Common Standards, Criteria, Indicators, and Evidence of Compliance on the different domains including Healthcare Provider (Individual-based and Population-based Services), Educator, Coordinator, Research, Navigator, and Leader/Manager (16).

The program’s commitment to continuous improvement culminated in March 2025, when it was awarded Level 3 (Full) Program Accreditation by the PAFP, signifying its full compliance with national standards and its readiness to sustain high-quality training in the years ahead.

A thematic analysis (Table 2) of the three accreditation reports from 2021 to 2024 provided a comprehensive view of the program’s evolution from its inception to its current status. The program evaluation reports which utilized the CIPP (Context, Input, Process, Product) Model (17) documented how the program yielded substantial outputs and outcomes in all domains.

**Table 2:** Thematic analysis summary of the program evaluation reports of the PAFP residency accreditation board on the Sorsogon Province-wide PBFCMRTP.

C-I-P-P Dimensions	Strengths	Areas for Improvement
Context	• Strategic alignment with UHC Law provisions • Integration with the Province HCPN and SDN • Strong LGU support and stakeholder partnership• Flexibility in training design to accommodate learners’ needs and training locale context	• Need to develop more structured tools to evaluate trainees’ activities in practice sites • Further define promotion criteria and process of assessment
Input	• Contextualized curriculum • Distributed learning sites • Use of ICT (digital platforms)• Committee faculty • Use of mentoring	• Need to develop a recruitment and capacity building plan for local faculty to supplant the inadequate number of trainers
Process	• Blended learning strategies • Structured mentoring sessions • Modular approach • Integrated case discussions	• Need to improve family focused care • Need to augment procedural skills training • Need to improve evaluation of learning outcomes vis health system improvements
Product	• Increased clinical competence of trainees • Professional identity formation • PHC approach and systems thinking application	• Need to manage trainee attrition due to competing workload and responsibilities

During this short period of implementation, the program contributed to improvements in service delivery through gatekeeping efficiency and care continuity. Residents developed competencies in system navigation, quality improvement, and interdisciplinary collaboration—skills that are critical to achieving Universal Health Coverage.

From the perspective of resident trainees, the program delivered more than technical skills—it fostered a deep transformation in clinical identity, systems engagement, and professional values. Residents reported that the curriculum’s alignment with the PFC model enabled a shift from symptom-based treatment to holistic, systems-informed care. They developed greater confidence in managing chronic conditions, applying clinical guidelines, and coordinating across service levels—core competencies aligned with UHC goals.

Trainees credited the hybrid, flexible learning format and mentorship culture as key growth enablers. The program’s hybrid learning approach proved essential in bridging geographic barriers while promoting reflective practice. Mentorship emerged as a central enabler, offering both professional guidance and psychosocial support. Mentoring sessions not only guided clinical practice but also supported personal development and career resilience.

Notably, structured assessments such as integrated case discussions, census audits, and Evidence Based Medicine (EBM) appraisals reinforced reflective practice and quality improvement. These tools helped residents understand the interplay between evidence-based care and real-world challenges, particularly in resource-constrained rural settings. These findings are consistent with global evidence highlighting the value of distributed training in building socially accountable, community-responsive health professionals (10).

A particularly striking outcome was the transformation in professional mindset and identity. Trainees began to view themselves not only as care providers but also as educators, leaders, researchers, and system navigators. The six roles of the Filipino family physician were internalized through practice, mentorship, and guided reflection. Longitudinal narrative accounts revealed how trainees evolved into confident, community-rooted physicians capable of advocacy, leadership, and systems-level change.

The program also promoted systems thinking and proactivity. Residents began initiating care coordination across service levels, identifying gaps, and proposing solutions. For example, trainees described how they improved hospital discharge protocols and strengthened referral loops—actions critical to the functioning of an integrated health provider network.

These outcomes affirm the strength of the distributed training model in cultivating not only technical competence but also values-driven leadership. The Sorsogon experience suggests that training in place, when structured through mentorship and aligned with health system goals, can produce a rural health workforce that is both clinically skilled and socially responsive. The program has also contributed to recruitment and rural physician retention in Sorsogon.

### Lessons learned

The implementation of the Sorsogon Province-wide PBFCMRTP offers valuable insights for enhancing distributed training in rural health systems, particularly in LMICs. The lessons learned can be categorized into facilitative strategies, challenges, and opportunities for growth, based on 4 years of program experience and qualitative feedback from stakeholders.

#### Facilitative strategies and enabling factors

##### Local ownership and good governance drive sustainability

One of the most critical enablers of success was the strong ownership of the program by the Provincial Government of Sorsogon. By embedding the training model within the province’s service delivery and health workforce development strategies, the program gained political support, financial commitment, and integration with local health system priorities. This decentralized governance structure allowed for greater responsiveness to contextual realities, such as staffing shortages and local health needs.

*“The presence of training residents in rural health units brought renewed energy to service delivery,”* noted the Provincial Health Officer, reinforcing the value of embedding training in service.

##### Mentorship is foundational for trainee retention and competency development

Structured, longitudinal mentorship emerged as a cornerstone of the program’s success. Each trainee was paired with an accredited family medicine mentor who provided clinical guidance, psychosocial support, and reflective learning opportunities. This mentoring relationship proved particularly important in building confidence, sustaining motivation, and preventing professional isolation—common barriers in rural postings.

As one resident reflected, *“Mentorship helped me reflect, reset, and grow into the doctor I wanted to be.”*

The program’s mentorship model compensated for limited faculty availability by leveraging remote supervision and group mentoring, which were highly valued by trainees.

##### Fit-for-purpose curriculum reflected local needs and system realities

A major strength of the PBFCMRTP was its curriculum design, which was context-specific, resource-sensitive, and aligned with both national accreditation standards and the provincial service delivery network. Trainees reported that the curriculum’s emphasis on the PFC model improved their ability to manage real-world cases, navigate referral systems, and deliver holistic care.

The use of the ICR method helped assess not only clinical competence but also the application of systems thinking and health promotion in routine practice.

##### Blended learning and digital tools can overcome geographic and lack of faculty

The hybrid learning approach—including virtual classrooms, asynchronous modules, and on-site mentoring—was instrumental in maintaining training continuity without pulling physicians away from their service sites. This flexibility enabled learners to manage real patient loads while progressing through structured competency development.

*“I could learn while staying in my municipality—this kept services going,”* shared one trainee.

The strategic use of digital platforms allowed limited faculty to support multiple learners across remote locations, improving training efficiency.

##### Accreditation can be used as a tool for program strengthening

Rather than treating accreditation as a bureaucratic hurdle, the program used it as a developmental benchmark. Over 4 years, the program progressed from “New Program” to Level 3 full accreditation by the PAFP, with iterative revisions made in response to formal feedback.

This outcome affirms that distributed training models can meet national standards for residency education when supported by structured evaluation and ongoing quality improvement.

#### Challenges encountered

Despite these successes, the province-wide residency training program faces significant implementation challenges. Foremost among them are the critical shortage of PAFP-certified faculty, inadequate diagnostic and training infrastructure in certain rural locations, and the lack of sustainable financing mechanisms for program expansion and faculty development. These constraints highlight the urgent need for sustained investment in rural teaching capacity and a more robust integration with national health workforce development strategies to ensure the program’s effectiveness and sustainability.

#### Opportunities for growth and innovation

The province-wide practice-based family and community residency training program presents several promising opportunities for growth, particularly through its ongoing curriculum revisions—four in total since its inception. These revisions have been instrumental in enhancing the training experience for resident trainees, allowing for a more comprehensive and relevant training framework. One notable opportunity is the incorporation of a rural surgery program, which offers residents the chance to gain practical skills within a limited scope of surgical practice tailored to the unique needs of the province. This initiative not only equips trainees with essential surgical competencies but also addresses the healthcare gaps in underserved areas, thereby fostering a more robust healthcare workforce. By continuing to adapt and respond to the evolving healthcare in the province, the program can further strengthen its impact and better prepare residents for their roles in family and community health.

The Sorsogon Province-wide PBFCMRTP provides a compelling model for addressing health workforce inequities in LMICs through distributed, community-embedded training. By leveraging local ownership, structured mentorship, and a context-specific curriculum, the program successfully aligns training goals with service delivery needs, enhancing both trainee competency and rural health system foundations. Despite challenges such as faculty shortages and infrastructure limitations, the program’s adaptive strategies and continuous improvements present significant opportunities for growth, particularly through curriculum enhancements and rural surgery initiatives. Overall, the program demonstrates that with strategic alignment and sustained investment, scalable solutions for rural health workforce development are achievable.

## Conclusion

The Sorsogon Province-wide PBFCMRTP demonstrates that distributed, community-embedded training models can effectively strengthen the rural primary care workforce in LMCIs. By aligning medical education with local health system needs, the program addresses persistent gaps in access, equity, and workforce retention without compromising service delivery.

The program’s fit-for-purpose curriculum, anchored in the PFC model, produced physicians capable of navigating complex care environments, coordinating across service levels, and leading health systems improvement initiatives in their communities. Through strategic use of mentorship, hybrid learning platforms, and provincial government ownership, the program successfully integrated residency training into the local healthcare ecosystem—resulting in improved clinical competencies, increased retention, and strengthened health systems performance.

The Sorsogon Province-wide PBFCMRTP confirms that residency training does not need to be centralized or hospital-bound to be rigorous and impactful. When designed for context, grounded in community realities, and supported by enabling policies and partnerships, distributed training models can serve as powerful instruments for rural health systems transformation. The lessons from Sorsogon provide a compelling case for replication in other provinces in the Philippines and similarly situated LMICs working toward UHC Coverage.

### Acknowledgement of any conceptual or methodological constraints or limitations

This community case study reflects the unique context of a provincial health system in the Philippines and is not intended to be generalized across all rural training environments. The findings and lessons are drawn from a single implementation site at Sorsogon province, where enabling conditions such as strong local government support, a motivated pool of trainees, and an existing partnership with the PAFP contributed to the program’s early success. As such, replication in other settings may require adaptation to differing governance capacities, workforce compositions, and infrastructure readiness.

Methodologically, the study relied on qualitative evaluation through document analysis, accreditation reports, and narrative reflections by trainees and mentors. While this approach allows for rich, context-sensitive insights, it may be limited by subjectivity and potential reporting bias. Quantitative measures such as patient outcomes, service utilization patterns, or long-term workforce retention were not systematically collected at this stage and will be important to include in future studies to strengthen impact attribution.

Additionally, the study only captures outcomes from the first 4 years of implementation. Longitudinal evaluation will be needed to assess sustained effects on health system performance, trainee career trajectories, and population health outcomes. Despite these limitations, the Sorsogon Province-wide PBFCMRTP offers a grounded example of how distributed training can be implemented effectively in resource-constrained settings, with meaningful implications for policy and practice.

## Data Availability

The raw data supporting the conclusions of this article will be made available by the authors, without undue reservation.
